# Genetically predicted serum metabolites mediate the association between inflammatory proteins and polycystic ovary syndrome: a Mendelian randomization study

**DOI:** 10.3389/fmed.2024.1433612

**Published:** 2024-12-03

**Authors:** Ming-Jie Jia, Li Zhou, Xing-Ning Liu, Hui-Lin Li

**Affiliations:** ^1^The Fourth Clinical Medical College of Guangzhou University of Chinese Medicine, Shenzhen, China; ^2^Institute of Depression and Comorbidity, Nanjing University of Chinese Medicine, Nanjing, Jiangsu, China; ^3^Shenzhen Traditional Chinese Medicine Hospital, Shenzhen, Guangdong, China

**Keywords:** Mendelian randomization, inflammatory proteins, serum metabolites, polycystic ovary syndrome, Interferon gamma, Chemokine C-C Motif Ligand 7, Interleukin-6, Matrix metalloproteinase-10

## Abstract

**Objective:**

To investigate the association between polycystic ovary syndrome (PCOS) and inflammatory proteins, and to identify and quantify the role of serum metabolites as potential mediators.

**Methods:**

Utilizing summary-level data from a genome-wide association study (GWAS), we conducted a two-sample Mendelian Randomization (MR) analysis, a genetic approach that uses genetic variants as instrumental variables to assess the causal relationships between risk factors and outcomes. This analysis involved genetically predicted PCOS (1,639 cases and 218,970 controls) and inflammatory proteins (14,824 participants of primarily European descent). Additionally, a two-step MR analysis was performed to quantify the proportion of the effect of serum metabolites-mediated inflammatory proteins on PCOS. The Inverse Variance Weighted (IVW) method, a statistical technique used within MR to combine data from multiple genetic variants, was used to estimate the causal effects.

**Results:**

The IVW method revealed that the inflammatory proteins IFN-γ (*p*-value = 0.037, OR = 1.396, 95% CI = 1.020–1.910) and CCL7 (*p*-value = 0.033, OR = 1.294, 95% CI = 1.021–1.641) were associated with an increased risk of PCOS, while IL-6 (*p*-value = 0.015, OR = 0.678, 95% CI = 0.495–0.929) and MMP-10 (*p*-value = 0.025, OR = 0.753, 95% CI = 0.587–0.967) were associated with a decreased risk. No significant evidence suggested an effect of genetically predicted PCOS on inflammatory proteins. The serum metabolite X-11444 was found to mediate 5.44% (95% CI: 10.8–0.0383%) of the effect of MMP-10 on PCOS.

**Conclusion:**

This study not only introduces novel causal associations between inflammatory proteins and PCOS but also highlights the mediating role of serum metabolites in these associations. By applying MR, we were able to minimize confounding and reverse causality, offering robust insights into the biological mechanisms underlying PCOS. These findings advance the understanding of PCOS pathogenesis, particularly in relation to inflammatory pathways and serum metabolite interactions, and suggest potential therapeutic targets that could inform future clinical interventions aimed at mitigating inflammation-related PCOS risks.

## 1 Introduction

Polycystic ovary syndrome (PCOS) is a prevalent endocrine disorder among women of reproductive age, characterized by metabolic and hormonal disturbances. One of the key diagnostic criteria for PCOS is ovulatory dysfunction, which typically presents as irregular or infrequent menstruation (1). This menstrual irregularity is often associated with underlying hormonal imbalances, particularly related to the excess production of androgens. Alongside this, hyperandrogenism frequently occurs, which manifests as hirsutism, acne, and other androgen-related symptoms. A polycystic ovarian morphology, observed on ultrasound, characterized by enlarged ovaries with multiple small follicles, is another key feature but is not required for diagnosis (2). With the increasing recognition of lifestyle factors such as sedentary behaviors and environmental influences, PCOS is not only a reproductive health concern but also significantly increases the risk of developing chronic conditions such as type 2 diabetes, cardiovascular diseases, and stroke (3). Identifying modifiable risk factors and understanding the underlying mechanisms of PCOS are crucial steps toward improving prevention and management strategies. Timely medical and lifestyle interventions can play a significant role in mitigating its effects.

Inflammatory and immune responses play a crucial role in the progression of PCOS. Studies have shown that inflammatory markers are significantly elevated in women with PCOS compared to healthy controls. Elevated levels of pro-inflammatory cytokines such as Interleukin-6 (IL-6) and tumor necrosis factor-alpha (TNF-α) are implicated in insulin resistance by disrupting the insulin signaling pathway (4). Inflammation also contributes to ovarian dysfunction, affecting the normal growth and release of follicles in PCOS patients (5). Reducing inflammatory responses has been shown to improve metabolic outcomes and reproductive health in patients with PCOS (6, 7). Macrophage dysfunction exacerbates inflammation, leading to ovarian damage and further compromising fertility (8). Effective anti-inflammatory treatments can significantly enhance insulin sensitivity in PCOS patients, providing a promising therapeutic avenue (9). Inflammation also affects key insulin target tissues, such as adipose tissue, liver, and muscle, contributing to metabolic dysfunction seen in PCOS (10). Therefore, investigating circulating inflammatory proteins could pave the way for novel preventive and therapeutic strategies at the molecular level. Numerous observational studies have established a strong association between circulating inflammatory factors and PCOS (11, 12). However, the cross-sectional nature of these studies introduces potential biases, such as confounding and reverse causation, which hinder accurate causal inference. Advances in proteomics, alongside large-scale genomic data integration, have significantly improved our ability to explore the mechanisms underlying complex diseases, particularly in understanding the genetic architecture of circulating proteins (13). Studying the genetic factors associated with inflammation-related proteins may provide new insights into the pathophysiology of PCOS and its complications (14).

Mendelian Randomization (MR) has emerged as a valuable tool for elucidating causal relationships between risk factors and health outcomes, utilizing genetic variants as instrumental variables. MR mimics the design of randomized controlled trials (RCTs) by capitalizing on the random assortment of alleles during gametogenesis, thereby minimizing confounding biases inherent in conventional observational studies (15). By leveraging genetic variants as instrumental variables, MR provides a robust framework for minimizing confounding and reverse causation, making it superior to traditional observational studies in establishing causal relationships. Previous studies have applied MR to investigate the relationship between specific diseases, such as type 2 diabetes and its complications, and certain pro-inflammatory cytokines (16). However, the role of circulating inflammatory proteins in PCOS remains understudied.

Our study employs a novel two-step MR approach to investigate the complex interplay between inflammatory proteins, serum metabolites, and PCOS. PCOS is characterized by metabolic and inflammatory disturbances, including energy and lipid metabolism disorders, which are associated with insulin resistance, obesity, and other features of metabolic syndrome (17). Alterations in amino acid metabolism also contribute to hormonal imbalances and metabolic homeostasis, while inflammatory pathways are linked to the chronic inflammatory state observed in PCOS patients (18). By integrating bidirectional and two-step MR designs, we aim to evaluate both the direct and mediated effects of 91 circulating inflammatory proteins and 1,400 serum metabolites using genome-wide association data (GWAS). This comprehensive approach allows us to explore how these biological pathways contribute to the pathogenesis of PCOS, thereby enhancing our understanding of its metabolic and inflammatory mechanisms. Unlike traditional MR analyses, our method incorporates serum metabolites as mediators, revealing their role in linking inflammation to PCOS. These insights could inform the development of targeted therapies to modulate specific inflammatory and metabolic pathways, paving the way for improved personalized treatment strategies. Additionally, our approach introduces a robust framework for causal inference in genetic epidemiology that can be applied to other complex diseases, enhancing the precision of causal estimates and minimizing biases such as confounding and pleiotropy.

## 2 Methods

**Figure 1:**
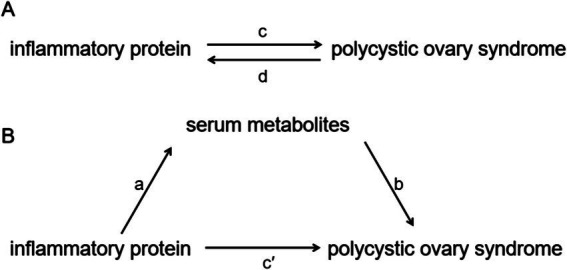
Diagrams illustrating the associations examined in this study. **(A)** Total effect between inflammatory proteins and PCOS. Parameter c represents the total effect of genetically predicted inflammatory proteins on PCOS, while d represents the total effect of genetically predicted PCOS on inflammatory proteins. **(B)** The total effect is decomposed into: (i) the indirect effect, assessed via a two-step approach, where a denotes the effect of inflammatory proteins on serum metabolites, and b denotes the effect of serum metabolites on PCOS, with the product method (a × b) capturing this indirect pathway; and (ii) the direct effect, calculated as c′ = c – a × b. The proportion mediated was determined by dividing the indirect effect by the total effect.

MR analysis relies on three key assumptions: (I) instrumental variables (IVs), which are genetic variants that act as proxies for the exposure of interest (inflammatory proteins), must be strongly associated with the exposure; (II) the IVs should be independent of confounding factors, meaning they are not related to any other variables that could influence the outcome (PCOS); and (III) the IVs must influence the outcome solely through the exposure, rather than through any alternative pathways. Our analytical workflow is depicted in Figure 1. We applied a two-sample bidirectional MR approach to evaluate the causal relationships between inflammatory proteins and PCOS, as shown in Figure 1A, to capture the total effect. Data on 91 circulating inflammatory proteins from a recent comprehensive study were selected as exposure factors, and rigorous criteria were applied to identify appropriate IVs. Using data from two GWAS, we conducted bidirectional MR analyses to investigate potential causal relationships between inflammatory proteins and PCOS.

The data used in this study were obtained from previous research, which had been approved by relevant ethics committees, with all participants providing informed consent. Therefore, additional institutional review board approval was not required.

### 2.1 GWAS summary data sources

We utilized revised GWAS summary data from 91 inflammatory proteins derived from Zhao et al. (14), involving 14,824 participants of predominantly European descent from 11 cohorts. The investigators measured genome-wide genetic and plasma proteomic data using Olink targeted inflammation and immunity assay panels, with concentrations of inflammatory proteins calculated. Inclusion criteria for this dataset required participants to be free from major inflammatory or autoimmune conditions that could bias inflammation-related analyses. Participants were also required to provide informed consent and meet genetic quality control criteria, such as passing Hardy–Weinberg equilibrium tests and maintaining a high genotype call rate. For each cohort, GWAS analyses used a linear regression-based additive genetic association model, reporting the effects of inflammatory proteins as inverse rank-normalized changes in protein concentrations per effect allele dose. To account for population stratification, genetic principal components were incorporated into the analyses, with age and sex included as covariates to minimize confounding factors. Detailed descriptions of quality control processes and data sources are available in the primary publication (14).

PCOS summary data were obtained from a GWAS by the FinnGen consortium (version R10) (19), which involved individuals of European ancestry (Table 1). The diagnostic criteria of PCOS were based on ICD-9 and ICD-10 standards (presence of two of the three criteria: chronic anovulation, hyperandrogenism, polycystic ovaries on ultrasonography). Participants were excluded if they had missing genetic information, failed quality control measures (such as low genotype call rates or deviations from Hardy–Weinberg equilibrium), or were of non-European ancestry. This ensured consistency in the analysis and minimized bias due to population stratification.

**Table 1:** GWAS data source for PCOS.

Disease categories	Phenocode	Ancestry	num_cases	num_controls
Polycystic ovarian syndrome	E4_PCOS	European	1,639	218,970

The GWAS summary statistics for 1,400 serum metabolites were obtained from research by Chen et al. (20), which involved a comprehensive analysis of 8,299 participants from the Canadian Longitudinal Study on Aging (CLSA) cohort. This study included 1,091 metabolites and 309 metabolite ratios, using genetic signals linked to known genes to assess the causal effects of metabolite levels and ratios on 12 traits and diseases primarily related to aging, metabolism, and immune response. Due to the complex nature of PCOS, which involves both metabolic and inflammatory disturbances, we adopted a hypothesis-free approach that included all available metabolites from the GWAS dataset. This study selected serum metabolites based on the available GWAS database without pre-screening for specific metabolites. We used a MR approach to comprehensively explore the potential association of all available metabolites with PCOS. This hypothesis-free approach ensured the breadth of the analysis and avoided selection bias. The complete GWAS summary statistics are available in the NHGRI-EBI GWAS Catalog at https://www.ebi.ac.uk/gwas/, under the European GWAS accession number GCST90199621-902010209.

To minimize bias from population stratification, we restricted our analysis to individuals of European ancestry. This decision was made to reduce confounding that may arise from differences in allele frequencies across populations of different ancestries. Population stratification can introduce bias into genetic association studies, leading to false-positive or false-negative results, especially in analyses involving complex traits such as PCOS. By focusing on a genetically homogeneous group, we aimed to increase the reliability of our findings. However, we acknowledge that this restriction limits the generalizability of our results to non-European populations. The differences in genetic architecture across populations mean that the associations we identified in individuals of European descent may not necessarily apply to other ethnic groups. Therefore, further studies involving diverse populations are necessary to confirm these findings and explore the role of inflammatory proteins and serum metabolites in PCOS across different ethnicities.

### 2.2 Selection of instrumental variables

To ensure the reliability of IVs, we applied a rigorous quality control process for single nucleotide polymorphisms (SNPs). First, we identified SNPs associated with inflammatory proteins that met the genome-wide significance threshold (*p*-value < 5 × 10^−6^), ensuring that the IVs accurately represented the exposure factors (21). Second, we used the European population reference panel from the 1,000 Genomes Project, accessed via the OpenGWAS API^1^ (22). A clustering method based on linkage disequilibrium (*R*^2^ < 0.001 within a 10,000 kb window) ensured IV independence. Third, we conducted a search in the Ensemble database to exclude SNPs that might violate the core assumptions of MR (23), thereby reducing potential biases from confounding factors or horizontal pleiotropy. To avoid erroneous allele assignments and accurately evaluate causal relationships, palindromic SNPs with uncertain strands and SNPs with discrepant alleles were excluded. Additionally, we calculated the F-statistic for each instrumental variable to assess its predictive strength (*R*^2^ = 2 × EAF × (1−EAF) × β^2^; *F* = *R*^2^ × (*N*−2)/(1−*R*^2^)) (24, 25). The F-statistic is used to test how strongly a genetic variant (SNP) is associated with the exposure. A higher F-statistic indicates a stronger association, which is crucial to avoid weak instrument bias. Although the two-sample MR method is a powerful tool for causal inference, it can be susceptible to weak instrument bias when the association between genetic variants and exposure is not strong. To address this, we ensured that all selected SNPs had F-statistics greater than 10, indicating sufficient instrument strength. Based on these criteria, unsuitable instrumental variables were excluded, and comprehensive measures were employed to ensure the precision and reliability of our results.

### 2.3 Statistical analysis

#### 2.3.1 Mendelian randomization

We used the MR method to investigate the association between circulating inflammatory proteins and PCOS. MR is a statistical approach that uses genetic variants as IVs to determine whether an exposure (inflammatory proteins) has a causal effect on an outcome (PCOS). This method mimics the design of randomized controlled trials (RCTs) by relying on the random assortment of alleles during reproduction, which helps minimize confounding and reverse causality. The IVW method was selected as our primary analytical tool because it provides a weighted average of the causal estimates from different genetic variants, offering precise estimates of causal relationships. The initial analysis was conducted using the IVW method, where the Wald ratio estimates for each genetic variant were calculated and then combined using a multiplicative random-effects IVW model to account for potential heterogeneity among SNPs. Specifically, a random-effects IVW model was applied when heterogeneity was detected, while a fixed-effects IVW model was used when no heterogeneity was present. To further ensure the robustness of the results, we employed sensitivity analyses, including MR-Egger regression to detect horizontal pleiotropy, the weighted median method to provide robust causal estimates even when up to 50% of the genetic variants are invalid, and MR-PRESSO to identify and correct for potential outliers that could bias results due to pleiotropy.

Our study employs an advanced MR framework that incorporates both bidirectional and two-step MR designs to enhance causal inference. The bidirectional MR analysis was used to evaluate the possibility of reverse causation by treating PCOS as the exposure and inflammatory proteins as the outcome. This approach helps establish the directionality of observed associations and ensures the robustness of findings. Additionally, a two-step MR analysis was conducted to investigate the potential mediating role of serum metabolites in the relationship between inflammatory proteins and PCOS, allowing us to decompose the total effect into direct and indirect components. By integrating genetic data with proteomic and metabolomic information, this two-step MR approach offers novel insights into the biological pathways linking inflammation and PCOS. Through these innovations, our study provides a comprehensive methodological framework that captures the complex interactions between inflammation, metabolism, and reproductive health in PCOS. Such an approach not only strengthens causal inferences but also offers a more nuanced understanding of how specific inflammatory pathways may contribute to the development and progression of PCOS, thereby setting the groundwork for identifying potential therapeutic targets.

#### 2.3.2 Sensitive analysis

To address the possibility of bias due to horizontal pleiotropy, we conducted several sensitivity analyses. Horizontal pleiotropy occurs when a genetic variant influences the outcome through multiple pathways, not just through the exposure of interest (inflammatory proteins). This could bias the results of the MR analysis. Therefore, we used the following methods to evaluate and correct for pleiotropy: (i) MR-Egger: This method can detect and adjust for bias due to pleiotropy. MR-Egger regression provides an intercept term that can indicate the presence of pleiotropy. If the intercept is significantly different from zero, this suggests that horizontal pleiotropy may be present, and the causal estimate could be biased. (ii) Weighted median: This method provides a robust estimate of the causal effect, even if up to 50% of the genetic variants used as IVs are invalid. The weighted median method assigns weights to each genetic variant based on its precision, providing a more reliable estimate in cases where pleiotropy may affect some of the variants. (iii) MR-PRESSO (Mendelian Randomization Pleiotropy RESidual Sum and Outlier): This method is used to detect and correct for outliers in the data. MR-PRESSO performs a global test to detect horizontal pleiotropy and then identifies outliers, which are genetic variants that might be influencing the outcome through pathways other than the exposure. The removal of outliers helps to ensure that the MR analysis remains unbiased.

#### 2.3.3 Mediation analysis

**Figure 2:**
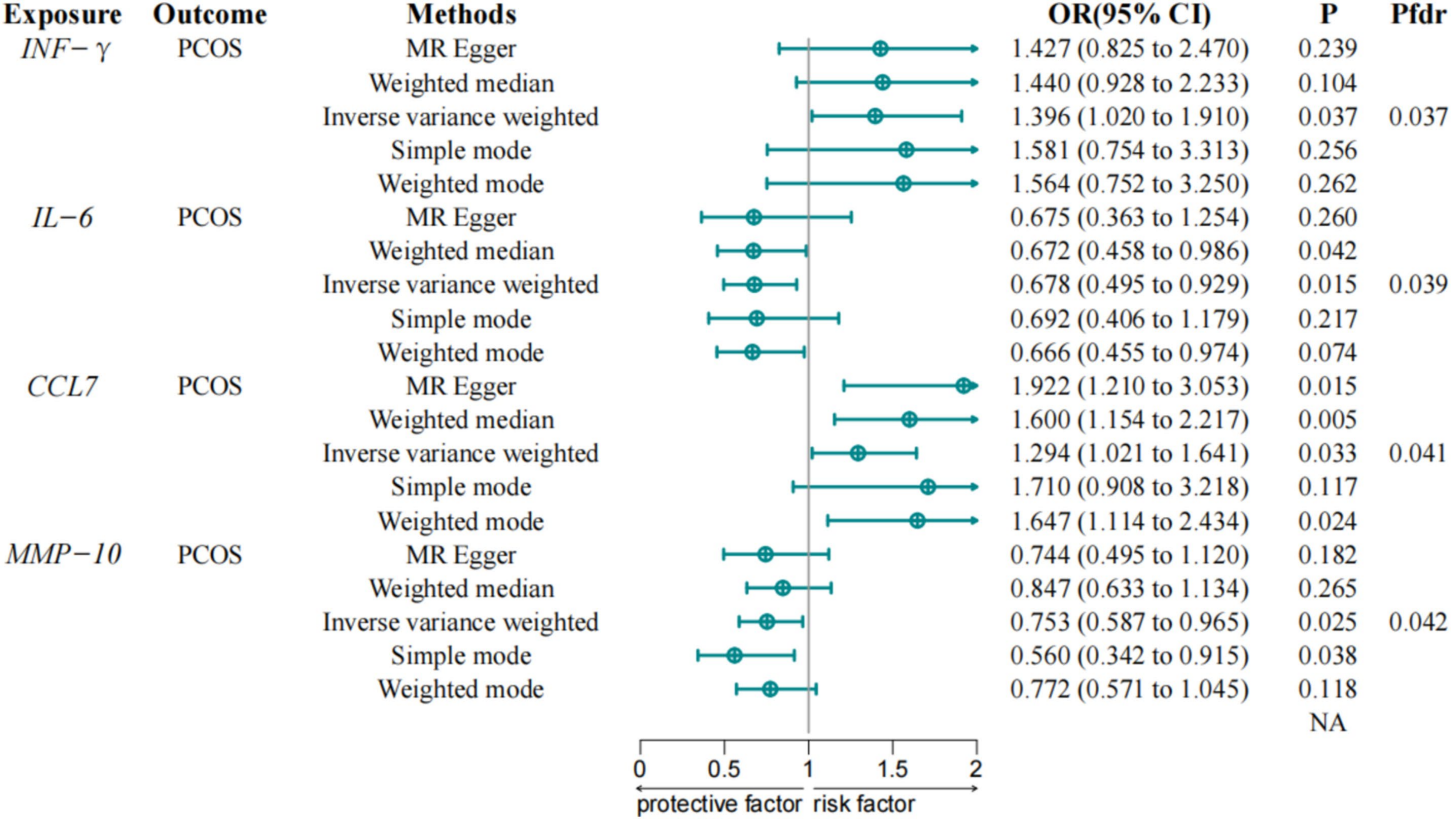
Associations between inflammatory proteins and PCOS in discovery cohorts. IVW, Inverse Variance Weighted; IFN-γ, Interferon gamma; CCL7, Chemokine C-C Motif Ligand 7; IL-6, Interleukin-6; MMP-10, Matrix metalloproteinase-10; PCOS, Polycystic Ovary Syndrome; FDR, False Discovery Rate.

A two-step MR design was employed to investigate whether serum metabolites mediate the causal pathway between inflammatory proteins and PCOS-related outcomes, as outlined in Figure 1B. The overall effect was partitioned into an indirect effect (mediated through serum metabolites) and a direct effect (independent of mediators) (21). Specifically, the total effects of inflammatory proteins on PCOS was divided into: (1) the direct effects of inflammatory proteins on PCOS (c’ in Figure 1B) and (2) the indirect effects mediated by inflammatory proteins through the metabolites (a × b in Figure 1B). The proportion mediated was quantified by calculating the ratio of the indirect effect to the total effect. Additionally, 95% confidence intervals for these estimates were calculated using the delta method (22).

## 3 Results

### 3.1 MR discovery analysis

Using the GWAS conducted by Zhao et al., we evaluated 91 inflammatory proteins for potential associations with PCOS using the IVW method with a multiplicative random effects model (Figure 2). The selection of these inflammatory proteins was based on a genome-wide significance threshold (*p* < 5 × 10^−6^), ensuring that the IVs for each protein met the assumptions required for causal inference in MR analysis. We applied stringent quality control measures to ensure the validity of the IVs, such as avoiding weak instruments (F-statistics > 10) and accounting for population stratification.

The IVW method analysis indicated that among the 91 proteins assessed, four proteins showed statistically significant associations with PCOS, including Interferon-gamma (IFN-γ) (OR = 1.396, 95% CI = 1.020–1.910, *p*-value = 0.037) and Chemokine C-C Motif Ligand 7 (CCL7) (OR = 1.294, 95% CI = 1.021–1.641, *p*-value = 0.033), which were associated with an increased risk of PCOS, while IL-6 (OR = 0.678, 95% CI = 0.495–0.929, *p*-value = 0.015) and Matrix metalloproteinase-10 (MMP-10) (OR = 0.753, 95% CI = 0.587–0.967, *p*-value = 0.025) were associated with a reduced risk of PCOS. These associations maintain statistical significance following correction for the FDR. A detailed report of all inflammatory proteins assessed, including those that did not show significant associations with PCOS, is provided in Supplementary file S1 to ensure completeness and transparency.

Sensitivity analyses revealed no evidence of pleiotropy bias in the causal estimates. The MR-Egger intercept analysis showed no directional pleiotropy between inflammatory proteins and PCOS, while Cochran’s Q test detected no significant heterogeneity (*p* > 0.05). The leave-one-out test identified no outliers, ensuring the robustness of the results, and the MR Steiger directional test detected no anomalies.

### 3.2 Reverse MR analysis

To explore the possibility of reverse association, we conducted an MR analysis treating PCOS as the exposure and inflammatory proteins as the outcome. This analysis did not reveal any reverse relationships, suggesting that genetically predicted PCOS does not causally influence inflammatory proteins.

### 3.3 Association of serum metabolites with PCOS

**Figure 3:**
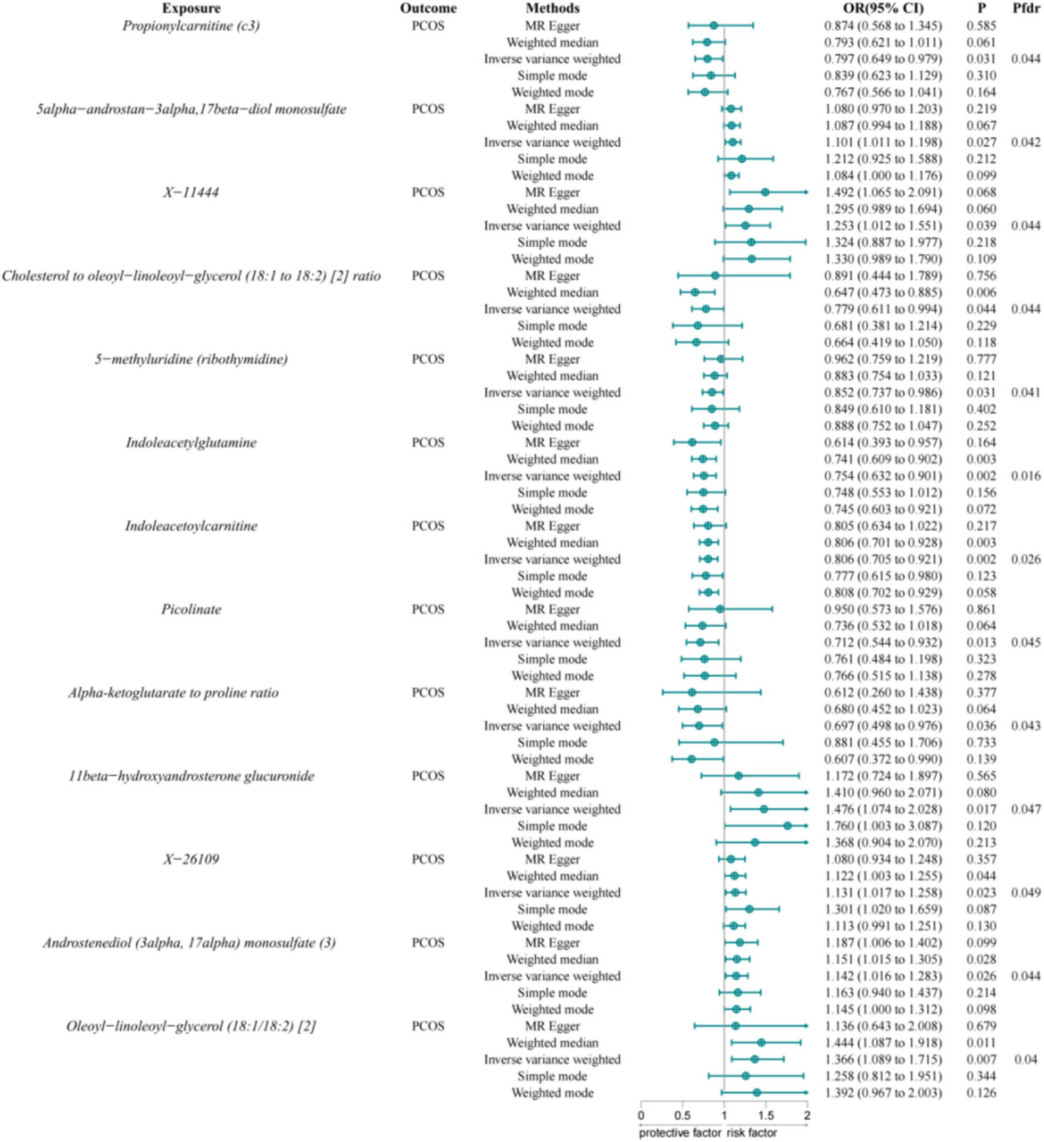
Associations between serum metabolites and PCOS. IVW, Inverse Variance Weighted; FDR, False Discovery Rate; PCOS, Polycystic Ovary Syndrome.

Using the IVW method and FDR correction, we evaluated 1,400 serum metabolites for potential associations with PCOS and identified 13 serum metabolites that were significantly associated with PCOS (Figure 3). Among these, Propionylcarnitine (C3) (OR = 0.797, 95% CI = 0.649–0.979, *p*-value = 0.031), Cholesterol to Oleoyl-Linoleoyl-Glycerol (18:1 to 18:2) ratio (OR = 0.779, 95% CI = 0.611–0.994, *p*-value = 0.044), 5-Methyluridine (ribothymidine) (OR = 0.852, 95% CI = 0.737–0.986, *p*-value = 0.031), Indoleacetylglutamine (OR = 0.754, 95% CI = 0.632–0.901, *p*-value = 0.002), Indoleacetylcarnitine (OR = 0.806, 95% CI = 0.705–0.921, *p*-value = 0.002), Picolinate (OR = 0.712, 95% CI = 0.544–0.932, *p*-value = 0.013), and Alpha-Ketoglutarate to Proline ratio (OR = 0.697, 95% CI = 0.498–0.976, *p*-value = 0.036) exhibited protective effects against PCOS. Conversely, 5alpha-androstan-3alpha, 17beta-diol monosulfate (1) (OR = 1.101, 95% CI = 1.011–1.198, *p*-value = 0.027), X-11444 (OR = 1.253, 95% CI = 1.012–1.551, *p*-value = 0.039), 11beta-Hydroxyandrosterone Glucuronide (OR = 1.476, 95% CI = 1.074–2.028, *p*-value = 0.017), X-26109 (OR = 1.131, 95% CI = 1.017–1.258, *p*-value = 0.023), Androstenediol (3alpha, 17alpha) monosulfate (3) (OR = 1.142, 95% CI = 1.016–1.283, *p*-value = 0.026), and Oleoyl-Linoleoyl-Glycerol (18:1/18:2) [2] (OR = 1.366, 95% CI = 1.089–1.715, *p*-value = 0.007) were associated with an increased risk of PCOS.

We now report the serum metabolites that showed significant associations with PCOS based on a significance threshold of *p* < 0.05. A full list of all metabolites assessed, including those that did not show significant associations, is provided in Supplementary file S2.

### 3.4 Association of inflammatory proteins with serum metabolites

Having established that four inflammatory proteins and 13 serum metabolites are critical for PCOS, we next investigated the causal impact of these proteins on the 13 serum metabolites. MR analysis revealed that IL-6 negatively affects 5alpha-androstan-3alpha, 17beta-diol monosulfate (1) (OR = 0.849, 95% CI = 0.742–0.973, *p*-value = 0.018). MMP-10 negatively affects X-11444 (OR = 0.934, 95% CI = 0.875–0.997, *p*-value = 0.039). CCL7 negatively affects Androstenediol (3alpha, 17alpha) monosulfate (3) (OR = 0.915, 95% CI = 0.840–0.998, *p*-value = 0.045).

### 3.5 Mediation effect of serum metabolites on PCOS

**Figure 4:**
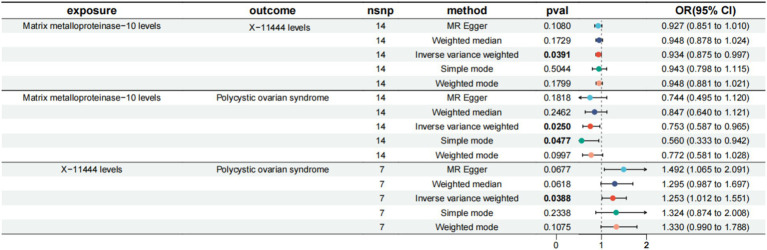
MR results of inflammatory factors and PCOS, serum metabolites and PCOS, inflammatory factors and serum metabolites. MMP-10, Matrix metalloproteinase-10; PCOS, Polycystic Ovary Syndrome; X-11444, Serum metabolite identifier; IVW, Inverse Variance Weighted; FDR, False Discovery Rate.

We assessed serum metabolites as intermediaries in the causal pathway from inflammatory proteins to PCOS (Figure 4). The mediating analysis revealed that X-11444 mediates 5.44% (95% CI = 10.8–0.0383%) of the total effect from MMP-10 to PCOS, with an indirect effect size of −0.0154 (95% CI = −0.0308, −0.000109, *p*-value = 0.048).

## 4 Discussion

In the past decade, a growing body of research has highlighted the pivotal role of inflammatory factors in the pathophysiology of various diseases, including PCOS. Numerous studies have suggested that inflammatory proteins contribute to the development and progression of PCOS, with elevated levels of pro-inflammatory cytokines like IL-6 and TNF-α reported in women with PCOS. Our study employs the MR approach to provide robust evidence of a causal relationship between these proteins and PCOS, reducing confounding and reverse causality, which are often present in traditional observational studies. The results of our MR analysis reveal significant relationships between four inflammatory proteins (IFN-γ, CCL7, IL-6, and MMP-10) and PCOS. To further explore these relationships, we conducted a two-step MR analysis to determine whether these proteins affect PCOS through serum metabolites. This analysis identified 13 serum metabolites, seven of which were associated with a protective effect against PCOS, while six were linked to an increased risk. We further quantified the indirect effects through mediation analysis, showing that certain serum metabolites mediate the effects of inflammatory proteins on PCOS. By including serum metabolites as mediators, our study provides a more nuanced understanding of the causal pathways linking inflammation to PCOS, offering insights into the mechanistic interactions and potential therapeutic targets.

Interferon-gamma (IFN-γ), a type II interferon primarily produced by natural killer (NK) and natural killer T (NKT) cells, plays a critical role in both innate and adaptive immunity. Beyond its antiviral and antitumor effects, IFN-γ significantly influences ovarian granulosa cell proliferation and apoptosis, potentially disrupting follicular development and ovulation, leading to reproductive dysfunction in PCOS patients (26–28). Observational studies have shown that elevated IFN-γ levels are linked to ovarian dysfunction, immune imbalance, and granulosa cell apoptosis in PCOS (29, 30). Moreover, in hyperandrogenemic conditions, high IFN-γ levels may enhance NK cell activity, further impairing fertility (31). Paravati’s study suggests that IFN-γ activates the Nuclear Factor Kappa-B (NF-κB) and Signal Transducer and Activator of Transcription 1 (STAT1) signaling pathways, resulting in abnormal expression of osteopontin and Cluster of Differentiation 44 (CD44) in the endometrium, which compromises endometrial receptivity and exacerbates reproductive dysfunction in PCOS (32). Similarly, Huang et al. demonstrated that elevated IFN-γ levels in PCOS mouse models induce granulosa cell apoptosis, macrophage pyroptosis, and impaired estrogen synthesis, highlighting its role in metabolic dysregulation (33). IFN-γ may also contribute to insulin resistance by reducing the expression of key proteins in the insulin signaling pathway, thereby promoting metabolic dysfunction in PCOS (34, 35). We extend the understanding of IFN-γ’s role beyond mere association, underscoring its potential as a therapeutic target. Our findings align with those of Paravati, suggesting that IFN-γ-driven activation of NF-κB and STAT1 contributes to endometrial dysfunction, and with Huang et al., who showed IFN-γ-induced metabolic disturbances and granulosa cell apoptosis. By providing causal evidence, our research enhances the understanding of IFN-γ’s multifaceted role in both the reproductive and metabolic dysfunctions of PCOS. The practical implications of our findings are significant. IFN-γ could serve as a valuable biomarker for identifying individuals at higher risk of developing PCOS, particularly those with chronic inflammation. Early detection of elevated IFN-γ levels could enable timely intervention to better manage PCOS symptoms. Furthermore, targeting IFN-γ or its downstream pathways could offer novel therapeutic approaches to reduce inflammation-related symptoms and improve reproductive and metabolic outcomes in PCOS patients. Future studies should explore the feasibility and efficacy of such targeted therapies, translating these insights into clinical benefits. In summary, our study demonstrates that IFN-γ levels are elevated in the secretory-phase endometrium of PCOS patients, indicating its key role in exacerbating chronic inflammation, insulin resistance, ovarian dysfunction, and endometrial impairment. These findings highlight the critical importance of IFN-γ in PCOS pathophysiology and emphasize the need for further research into therapeutic strategies targeting IFN-γ to improve the metabolic and reproductive health of PCOS patients.

Chemokine C-C Motif Ligand 7 (CCL7) also known as monocyte chemoattractant protein-3 (MCP-3), is a member of the CC chemokine subfamily. It primarily attracts monocytes and neutrophils, playing a significant role in various inflammatory processes. Studies show that in type 1 diabetes, CCL7 contributes to immune cells, aggravating β-cell destruction (36). In type 2 diabetes, CCL7 is associated with adipose tissue inflammation and insulin resistance, indicating its involvement in metabolic syndrome and diabetes development. Furthermore, increased CCL7 expression in the adipose tissue of obese and type 2 diabetic individuals correlates with macrophage infiltration and systemic inflammation, underscoring its importance in inflammation and insulin resistance in these conditions (37, 38). Given its role in modulating inflammatory responses and insulin sensitivity, CCL7 is hypothesized to similarly influence the pathophysiology of PCOS. CCL7 could intensify ovarian inflammation by attracting inflammatory cells, potentially impairing ovarian function and hormone balance (39). Recent findings have illustrated that CCL7 levels correlate with the degree of insulin resistance and the presence of metabolic syndrome in women with PCOS. This connection implies that CCL7 may serve as a critical link between inflammation and the metabolic features of PCOS, where elevated levels could exacerbate insulin resistance, thus further complicating the clinical picture of the disease (39). Understanding this relationship may provide insights into targeted therapies aimed at modulating CCL7 levels to improve metabolic outcomes in PCOS patients. This finding aligns with recent studies that have demonstrated elevated CCL7 levels in women with PCOS, linking it to increased ovarian inflammation and insulin resistance. Cavalcante et al. highlighted that higher levels of CCL7 are correlated with the severity of metabolic dysfunction in PCOS, reinforcing the significance of this inflammatory marker in the disease’s pathophysiology (40). Vasyukova et al.’s study indicated that MCP-3 levels are particularly elevated in lean PCOS patients, strongly suggesting a potential role for MCP-3 in the pathophysiology of PCOS (7). As an important chemokine, MCP-3 recruits monocytes and other immune cells to sites of inflammation, thereby amplifying inflammatory responses. Thus, we can reasonably speculate that elevated MCP-3 levels may directly contribute to the chronic low-grade inflammation observed in PCOS patients, which not only plays a role in ovarian dysfunction and ovulatory abnormalities but is also closely related to the occurrence of complications such as insulin resistance and metabolic dysregulation (11). Our findings align with these existing studies, emphasizing that elevated CCL7 levels contribute to the inflammatory and metabolic features of PCOS. While previous observational studies have established associations between CCL7 and metabolic disturbances, our study adds a new dimension by providing causal evidence through MR. This strengthens the understanding of CCL7’s role beyond correlation, highlighting its potential as a key factor in both the reproductive and metabolic aspects of PCOS. The consistency of our findings with existing literature supports the hypothesis that CCL7 is an important mediator in the inflammation-metabolism axis of PCOS. The practical implications of our findings are significant. Clinically, CCL7 may be a valuable biomarker for monitoring inflammatory status and metabolic health in PCOS patients. Early identification of elevated CCL7 levels could allow for timely intervention to manage PCOS symptoms more effectively, particularly for those at higher risk of developing severe metabolic complications. Furthermore, therapies aimed at reducing CCL7 levels or blocking its receptor could potentially mitigate the inflammatory and insulin-resistant aspects of PCOS, paving the way for novel treatment strategies. Such targeted approaches could improve both metabolic and reproductive outcomes in PCOS patients, offering a personalized treatment strategy based on individual inflammatory profiles. In summary, our study found that CCL7 levels are elevated in the secretory phase endometrium of PCOS patients, suggesting that it plays a key role in exacerbating chronic inflammation, insulin resistance, ovarian dysfunction, and endometrial dysfunction. Future research should explore the feasibility and efficacy of these targeted therapies to translate these insights into clinical benefits for PCOS patients.

Matrix metalloproteinases (MMPs) are zinc-dependent endopeptidases essential for extracellular matrix (ECM) degradation and remodeling, playing crucial roles in tissue repair, angiogenesis, and cell migration. Proper regulation of MMP activity is vital for maintaining tissue homeostasis, whereas dysregulation is linked to several pathological conditions, including cancer, cardiovascular diseases, and inflammatory disorders (41–43). In the context of PCOS, Matrix Metalloproteinase-2 (MMP-2) and Matrix Metalloproteinase-9 (MMP-9) have been implicated due to their contributions to ECM remodeling, inflammation, and tissue repair. Aberrant expression and increased activity of these MMPs can lead to fibrosis and ovarian tissue remodeling, exacerbating endocrine disruption and ovulation disorders (44). Together, MMP-2 and MMP-9 contribute significantly to the reproductive and metabolic dysfunctions observed in PCOS. Given the established roles of MMP-2 and MMP-9, MMP-10 is hypothesized to similarly influence PCOS pathogenesis. MMP-10, involved in ECM degradation and inflammatory regulation, could disrupt the ECM environment within ovarian follicles, impair ovarian angiogenesis, and potentially lead to cyst formation—a hallmark of PCOS (45–47). This dysregulated activity may further drive inflammation, hyperandrogenemia, and insulin resistance, complicating the metabolic profile of affected patients. Elevated levels of MMP-10 in other inflammatory and metabolic disorders also support its role in PCOS-related inflammation and metabolic dysregulation. Recent studies, such as those by Oktanella et al., reported elevated MMP-10 levels in PCOS patients, suggesting its potential as a biomarker for the disease (48). The dual impact of MMP-10 on inflammation and ECM remodeling makes it a promising therapeutic target for PCOS management. The findings in our study further support that MMP-10 may be a novel risk factor for PCOS, with a significant role in ECM degradation, ovarian angiogenesis, and inflammation. This underscores the need for further research to elucidate its mechanistic role and potential as a therapeutic target to address both metabolic and reproductive dysfunctions in PCOS.

Interleukin-6 (IL-6) is an inflammatory cytokine predominantly secreted by adipocytes and plays a key role in the pathophysiology of polycystic ovary syndrome (PCOS). Research consistently shows that serum IL-6 levels are significantly elevated in PCOS patients compared to healthy controls, correlating with the chronic low-grade inflammation characteristic of the condition (49, 50). IL-6 interferes with insulin signaling, contributing to insulin resistance and metabolic disorders related to glucose and lipid metabolism. Moreover, elevated IL-6 is associated with increased serum testosterone levels, irrespective of weight status, which further exacerbates the endocrine dysfunction seen in PCOS (51). Overproduction of IL-6 may also stimulate ovarian androgen biosynthesis, leading to irregular menstrual cycles and ovulatory dysfunction, making it a critical biomarker of inflammation and disease progression in PCOS. Elevated IL-6 can enhance the secretion of other inflammatory cytokines and chemokines, creating a vicious cycle of inflammation that perpetuates ovarian dysfunction. This highlights the interconnectedness of inflammatory pathways in PCOS, suggesting that comprehensive treatment may require targeting multiple inflammatory markers (51). Our findings are consistent with previous research, such as Başer et al.’s work, which demonstrated IL-6’s role in insulin resistance and supports the notion that inflammatory markers can serve as indicators of disease severity (52). Clinical significance of IL-6 in PCOS is profound: it not only serves as a biomarker for disease monitoring but also represents a potential therapeutic target. Targeting IL-6 with monoclonal antibodies or other inhibitors could reduce inflammation and improve metabolic profiles in PCOS patients, underscoring the need for further clinical exploration in this direction.

5α-androstan-3α, 17β-diol monosulfate is a significant androgen metabolite closely related to androsterone and stanolone (51, 53). Given its close association with androgens, it is reasonable to speculate that this metabolite may be involved in PCOS pathophysiology, considering the elevated androgen levels commonly observed in PCOS patients. However, studies have not identified significant differences in the levels of 5α-androstan-3α, 17β-diol monosulfate between PCOS patients and healthy individuals, suggesting it may not play a direct role in elevating androgen levels in PCOS. Nonetheless, this does not entirely exclude its potential involvement in local androgen signaling regulation. Further research is necessary to determine its role in PCOS, particularly in clinical symptoms and hormone regulation.

Chromium picolinate, with the chemical formula C18H12CrN3O6, has been studied for its potential benefits in PCOS patients. A systematic review and meta-analysis demonstrated that chromium picolinate supplementation could significantly reduce body mass index (BMI), fasting insulin, and free testosterone levels in PCOS patients, indicating improvements in metabolic disorders associated with the condition. Specifically, chromium picolinate was found to reduce BMI by 2.37 kg/m^2^, free testosterone levels by 0.52 pg./mL, and fasting insulin levels by 0.86 mIU/mL. However, no significant effect was observed on total testosterone, Ferriman-Gallwey (FG) score, dehydroepiandrosterone (DHEA), follicle-stimulating hormone (FSH), or luteinizing hormone (LH) levels (54, 55). These findings suggest that chromium picolinate may improve metabolic and endocrine outcomes, particularly in addressing insulin resistance and regulating testosterone levels. Nonetheless, further clinical trials are required to validate these results.

In light of recent research, the potential role of chromium picolinate in modulating inflammatory responses, particularly through reducing IL-6 levels, suggests that it could play a dual role in managing both metabolic and inflammatory aspects of PCOS. This multifaceted approach underscores the importance of integrating nutritional strategies into the overall management of PCOS, particularly for patients struggling with insulin resistance (56).

In the context of existing literature, our results on chromium picolinate align with findings from a systematic review by Fazelian et al. which also highlighted the benefits of chromium supplementation in improving metabolic parameters in PCOS patients (55). These findings support the notion that lifestyle and dietary interventions, including chromium supplementation, should be considered as part of a comprehensive management plan for PCOS. The potential for chromium picolinate to serve as a therapeutic adjunct in the treatment of PCOS-related metabolic dysfunctions merits further clinical trials to solidify its role.

α-ketoglutarate is an essential metabolite in various biochemical pathways, including antioxidation, energy production, and cellular signaling (57). It plays a key role in the citric acid cycle, maintaining energy balance and facilitating the production of glutamate and glutamine, two amino acids crucial for protein synthesis and muscle regulation. Given the metabolic disorders and oxidative stress commonly seen in PCOS patients, α-ketoglutarate’s antioxidative properties may help alleviate oxidative burden in these individuals. Moreover, α-ketoglutarate may enhance intestinal antioxidation, potentially boosting glutamine levels and strengthening antioxidative mechanisms. Although no direct evidence links α-ketoglutarate to PCOS, its involvement in energy metabolism and antioxidation suggests it could positively impact PCOS treatment.

Moreover, considering the inflammatory context of PCOS, α-ketoglutarate’s role as a precursor for the synthesis of key metabolites involved in the inflammatory response may also be crucial. Its potential to mitigate oxidative stress and enhance mitochondrial function could serve as a protective mechanism against the inflammatory damage seen in PCOS (58). This suggests that α-ketoglutarate supplementation might offer additional benefits by addressing both metabolic and inflammatory pathways in PCOS management. The potential benefits of α-ketoglutarate in PCOS have been mentioned in recent studies, which propose that it may aid in reducing oxidative stress and improving metabolic outcomes (58). Thus, α-ketoglutarate supplementation may represent a novel therapeutic strategy for managing oxidative stress and metabolic disturbances in PCOS patients, though clinical studies are needed to validate this approach.

11β-hydroxyandrosterone glucuronide is an endogenous metabolite produced when UDP-glucuronosyltransferase in the liver acts on 11β-hydroxyandrosterone. This biotransformation process, known as glucuronidation, is essential for the removal of toxic agents, medications, and other non-energy-providing substances from the body. During glucuronidation, glucuronic acid is conjugated to the parent compound through a glycosidic bond, resulting in glucuronide derivatives that are significantly more water-soluble, thereby facilitating their elimination via the kidneys (59). Research indicates that levels of 11β-hydroxyandrosterone glucuronide are markedly elevated in patients with PCOS compared to healthy individuals (60), suggesting that this metabolite may play a role in the pathophysiology of PCOS. Nonetheless, further studies are needed to elucidate the precise mechanisms and effects of 11β-hydroxyandrosterone glucuronide within the context of PCOS.

This elevation may indicate a dysregulated steroid metabolism, further compounding the hormonal imbalances commonly seen in PCOS. Investigating the pathways through which 11β-hydroxyandrosterone glucuronide influences ovarian function and inflammatory responses could provide valuable insights into its role in PCOS and lead to potential therapeutic interventions targeting this metabolite (60).

Studies have shown that the inflammatory response can vary significantly between women with different PCOS phenotypes, affecting the severity and type of symptoms experienced (61). It is worth noting that the phenotypes of PCOS are primarily based on the three core features defined by the Rotterdam criteria: oligo-ovulation or anovulation, hyperandrogenism, and polycystic ovarian morphology. Based on different combinations of these characteristics, PCOS is typically classified into four phenotypes: classic PCOS (meeting all three criteria), atypical PCOS (oligo-anovulation and hyperandrogenism without polycystic ovarian morphology), hidden PCOS (hyperandrogenism and polycystic ovarian morphology but with ovulation), and mild PCOS (oligo-anovulation and polycystic ovarian morphology without hyperandrogenism) (62). Although all phenotypes may exhibit ovulatory dysfunction leading to fertility issues, their clinical symptoms and metabolic risks differ. The presence of hyperandrogenism usually indicates a higher risk of metabolic complications, such as diabetes and cardiovascular disease, while phenotypes lacking hyperandrogenism have lower metabolic risks (63). Furthermore, some phenotypes, despite having polycystic ovarian morphology, may present relatively mild clinical symptoms, such as mild PCOS. The different phenotypes of PCOS may not only differ in pathological mechanisms but also influence the formulation of clinical intervention strategies. However, this study is limited by the availability of datasets and was unable to conduct stratified analyses of different PCOS phenotypes. This limitation restricts our ability to explore the complex interactions between inflammatory proteins and serum metabolites across the various phenotypes in depth. Future research should utilize more detailed and diverse databases to conduct phenotype stratification analyses to further reveal the specific mechanisms of different PCOS phenotypes and provide a basis for personalized treatment strategies. This also means that our conclusions primarily apply to the general PCOS population, and the specific differences between phenotypes require further validation.

The primary strength of our study lies in the application of the MR method, which minimizes the influence of reverse causality and confounding factors, allowing us to better establish the causal relationship between systemic inflammation modifiers and PCOS. Additionally, the study utilized the largest available GWAS dataset for PCOS and systemic inflammation, ensuring robust data collection and reliability of the findings. However, this study has several limitations. First, our focus on individuals of European ancestry may affect the generalizability of the findings. The genetic diversity across different populations can lead to variations in the prevalence and expression of inflammatory proteins, which may influence their association with PCOS. This suggests that the findings from our study may not be applicable to individuals from non-European backgrounds, potentially limiting the broader applicability of our conclusions. Moreover, environmental factors, lifestyle differences, and comorbid conditions prevalent in diverse populations could further affect the inflammatory response and the development of PCOS. Therefore, future studies should aim to include participants from various ethnic backgrounds to ensure a comprehensive understanding of how genetic and environmental factors interact to influence PCOS pathophysiology. Second, the lack of stratified analysis by PCOS phenotypes is a significant limitation. Due to the absence of phenotype-specific data, we were unable to assess the differences in these markers across various PCOS subtypes. Future research should include phenotypic stratification of PCOS patients to explore the specific roles of inflammatory proteins and metabolites in different PCOS phenotypes, which could help guide more targeted treatment approaches. Finally, while this study highlights potential associations between inflammatory proteins such as IFN-γ, CCL7, MMP-10, and IL-6 with PCOS, further research is needed to evaluate the clinical relevance of these markers. Future studies could assess whether these proteins could be used as biomarkers for early diagnosis or therapeutic targets, especially in PCOS patients with severe insulin resistance or metabolic dysregulation. The potential for these inflammatory markers to guide personalized treatment strategies in PCOS cannot be overstated; integrating biomarker assessment into clinical practice may enhance patient outcomes significantly. These inflammatory markers may offer new avenues for the clinical management of PCOS, particularly for patients who are unresponsive to traditional treatments and could benefit from personalized therapeutic strategies targeting specific inflammatory pathways. In conclusion, future research should expand to more diverse populations and incorporate stratified analysis by PCOS phenotypes to further deepen our understanding of the role of inflammatory proteins in PCOS prevention and treatment.

## 5 Conclusion

In conclusion, this study using the MR method demonstrates that inflammatory proteins and serum metabolites play crucial roles in PCOS pathophysiology. Markers like IFN-γ, CCL7, MMP-10, and IL-6 may serve as potential biomarkers or therapeutic targets, and clinicians could consider incorporating these markers into diagnostic protocols to identify PCOS patients with heightened inflammatory responses. These findings suggest that targeted interventions addressing specific inflammatory pathways could improve PCOS management, particularly for patients with severe metabolic or inflammatory profiles. Incorporating biomarkers identified in this study may enhance early diagnosis and personalized treatment strategies. While our study identified serum metabolite X-11444 as a potential mediator, there are currently no related studies reported. Future research should further validate this finding and explore its clinical significance. Additionally, future research should explore these associations in diverse populations and stratify analyses based on different PCOS phenotypes to tailor interventions effectively. Longitudinal studies are also needed to evaluate the long-term impact of targeting inflammatory pathways, as well as to determine the potential for these biomarkers to guide personalized treatments in PCOS and other related metabolic disorders.

## Data Availability

The original contributions presented in the study are included in the article/Supplementary material, further inquiries can be directed to the corresponding author.

## Footnotes

^1^ https://gwas-api.mrcieu.ac.uk/
